# Association of Urine sCD163 With Proliferative Lupus Nephritis, Fibrinoid Necrosis, Cellular Crescents and Intrarenal M2 Macrophages

**DOI:** 10.3389/fimmu.2020.00671

**Published:** 2020-04-15

**Authors:** Ting Zhang, Hao Li, Kamala Vanarsa, Gabriel Gidley, Chi Chiu Mok, Michelle Petri, Ramesh Saxena, Chandra Mohan

**Affiliations:** ^1^Department of Biomedical Engineering, University of Houston, Houston, TX, United States; ^2^Department of Medicine, Tuen Mun Hospital, Hong Kong, China; ^3^Division of Rheumatology, Johns Hopkins University School of Medicine, Baltimore, MD, United States; ^4^University Hospital Kidney & Liver Clinic, University of Texas Southwestern Medical Center, Dallas, TX, United States

**Keywords:** CD163, lupus nephritis, urine biomarker, renal pathology, activity index

## Abstract

CD163 is a marker for alternatively activated macrophages, which have been implicated in the pathogenesis of lupus nephritis (LN). In our preliminary screening of urine proteins in LN, urine soluble CD163 (sCD163) was significantly elevated in patients with active LN. To evaluate the potential of sCD163 as a biomarker in LN, urine sCD163 was assayed in patients with active LN, active non-renal lupus patients (ANR), inactive SLE and healthy controls (HC), using ELISA and normalized to urine creatinine. The correlation of urine sCD163 with clinical parameters and renal pathological attributes was further investigated in LN patients with concurrent renal biopsies. A total of 228 SLE patients and 56 HC were included from three cohorts. Results demonstrated that urine sCD163 was significantly elevated in active LN when compared with HC, inactive SLE, or ANR in African-American, Caucasian and Asian subjects (all *P* < 0.001). In LN patients with concurrent renal biopsies, urine sCD163 was significantly increased in patients with proliferative LN when compared with non-proliferative LN (*P* < 0.001). Urine sCD163 strongly correlated with SLEDAI, rSLEDAI, activity index (AI) of renal pathology, fibrinoid necrosis, cellular crescents, and interstitial inflammation on biopsies (all *P* < 0.01). Macrophages, particularly M2 macrophages, the predominant cells expressing CD163 within LN kidneys, represented a potential source of elevated urine sCD163, based on single-cell RNA sequencing analysis. To conclude, urine sCD163 discriminated patients with active LN from other SLE patients and was significantly elevated in proliferative LN. It strongly correlated with concurrent AI and several specific pathological attributes, demonstrating its potential in predicting renal pathology.

## Introduction

Systemic lupus erythematosus (SLE) is an autoimmune disease characterized by the production of autoantibodies and involvement of multiple organ systems. One of the leading causes of morbidity and mortality in SLE is lupus nephritis (LN), which is clinically evident in more than half of all SLE patients. Approximately 10–17% of LN patients will progress to end-stage renal disease (ESRD) (1–3). LN is classified by the International Society of Nephrology/Renal Pathology Society (ISN/RPS) into six categories based on pathological findings (4, 5). Class III and class IV, categorized as proliferative LN, are the most severe forms of LN, carrying the highest risk of progression to ESRD and thus requiring intensive immunosuppressive therapy (2, 6, 7).

Renal biopsy is the current gold standard for the diagnosis and classification of LN. However, the limited tissue obtained each time may not accurately reflect the complete spectrum of renal lesions in a given patient’s kidneys due to sampling error; moreover, its invasiveness and attendant complications discourage repeated biopsy at patient follow-up. In contrast, urine samples can be easily obtained and are ideal for frequent monitoring. Non-invasive urinary biomarkers may emerge as an alternative method for LN assessment, as these markers are more convenient to assay, one day even at home, and allow repeated examinations (8). Specific urinary biomarkers which discriminate active LN and predict concurrent underlying LN pathology could be instrumental in guiding the management of LN.

In our preliminary aptamer-based targeted proteomic screen of >1000 urine proteins in LN, urine CD163 was noted to be significantly higher in patients with active LN (Supplementary Figure 1). Here, we pursue this initial observation further, given that CD163 is a marker for alternatively activated macrophages, which have been implicated in the pathogenesis of LN (9). While CD163^+^ macrophages can only be observed and analyzed on tissue biopsies, soluble CD163 (sCD163), derived from the extracellular portion of CD163 when cleaved by metalloproteinases, can easily be measured in diverse body fluids, including serum, urine, synovial fluid and cerebrospinal fluid (9, 10). The shedding of CD163 is enhanced by various pathological conditions including infections, liver diseases, malignancies, and autoimmune diseases. Indeed, sCD163 has been used as a biomarker for macrophage activation in several inflammatory diseases (9, 11).

To evaluate the potential of urine sCD163 as a biomarker in LN, and to investigate its correlation with clinical indices and pathological attributes, we assayed urine sCD163 in SLE patients with diverse disease activities from multiple ethnicities.

## Materials and Methods

### Patients, Sample Collection, and Preparation

Urine samples from three cohorts of patients with SLE were included in this study. The primary cohort was comprised of 123 patients with SLE from the Division of Rheumatology, Johns Hopkins University (JHU) School of Medicine, Baltimore, MD, United States. The validation cohort included 60 patients with SLE from Tuen Mun Hospital, Hong Kong (HK). An independent cohort of 45 LN patients with concurrent renal biopsies was drawn from the University of Texas Southwestern (UTSW) Medical Center’s Renal Clinic, Dallas, TX, United States. Gender and ethnicity matched healthy subjects were recruited as controls. Informed consent was obtained from all patients, and this study was approved by the Institutional Review Board of the University of Houston, JHU School of Medicine, Tuen Mun Hospital, and UTSW. All patients met the 2012 Systemic Lupus International Collaborating Clinics (SLICC) classification criteria for SLE (12). For all patients, hybrid SLE disease activity index (SLEDAI) was used, where proteinuria was scored if > 0.5 g/24 h. The renal SLEDAI (rSLEDAI) summated the renal domains of SLEDAI, including hematuria (>5 red blood cells/high-power field), pyuria (>5 white blood cells/high-power field), proteinuria (>0.5 g/24h), and urinary casts. SLE patients were classified into three groups. Active LN (AR) was defined as biopsy-proven LN with rSLEDAI ≥ 4. None of the active LN patients in this study had isolated hematuria or pyuria. Active non-renal SLE patients (ANR) had SLEDAI ≥ 5 and rSLEDAI = 0. The Inactive (or low disease activity) group included SLE patients with SLEDAI ≤ 4, and clinical SLEDAI (omitting anti-dsDNA and complement) ≤ 2. Clean-catch midstream urine samples were collected and refrigerated within 1 hour of sample collection. The samples were then aliquoted and stored at −80°C. SLEDAI, rSLEDAI, physician global assessment (PGA), complete blood count, serum creatinine, urinalysis, urine protein to creatinine ratio, C3, C4, and anti-dsDNA were recorded.

### Renal Histology

In the UTSW biopsy-concurrent cohort of 45 LN patients, renal biopsies were performed within 30 days of urine procurement. All 45 renal biopsies were documented for LN classes, and 42 biopsies had been scored for activity index (AI), chronicity index (CI), and their component attributes. AI was scored based on 6 components including endocapillary hypercellularity, glomerular leukocyte infiltration, wire loop deposits, fibrinoid necrosis, cellular crescents, and interstitial inflammation. The CI was scored based on glomerulosclerosis, fibrous crescents, tubular atrophy and interstitial fibrosis. The maximum score was 24 points for the AI and 12 for CI (13).

### Assay of Urine sCD163 and Urine Creatinine

Urine sCD163 was assayed using a commercially available enzyme-linked immunosorbent assay (ELISA) kit (RayBiotech), following manufacturer instructions. Briefly, diluted urine samples were added to anti-CD163 pre-coated 96-well microplates, followed by biotin-conjugated anti-CD163 detection antibody, streptavidin-HRP, and substrate. Optical densities were read using a microplate reader at 490 nm (ELX808 from BioTek Instruments, Winooski, VT, United States). The levels of urine creatinine were assayed using the Creatinine Parameter Assay Kit (R&D Systems). Urine sCD163 values were normalized to urine creatinine before further analysis.

### Statistical Analysis

Data were analyzed using GraphPad Prism 7. The Mann Whitney *U* test was used for comparisons between two groups, and the analysis of variance (ANOVA) test with subsequent post-test pairwise comparisons was used for comparison of multiple groups. Chi-square test or Fisher’s exact test was used to compare percentages. Non-parametric Spearman correlation was performed for correlation analysis. Receiver operating characteristic (ROC) curve was used to compare the performance of urine sCD163 versus other parameters and to determine the optimal cut-off values. A two-tail *P* value less than 0.05 was considered significant.

## Results

### Urine sCD163 Was Significantly Elevated in Active LN in African Americans and Caucasians

**FIGURE 1:**
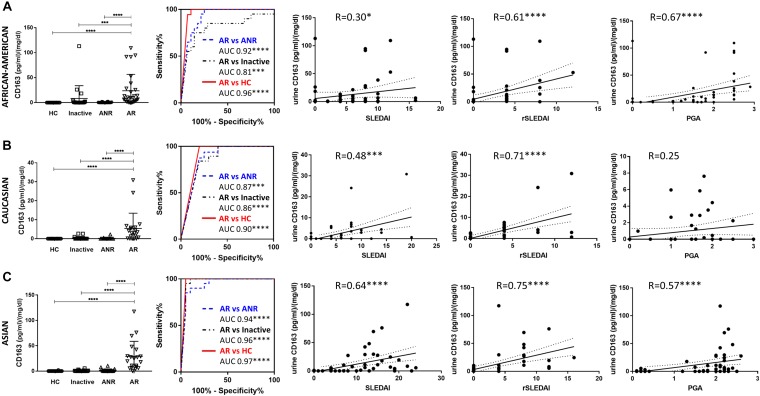
Urine sCD163 levels and its correlation with clinical indices. Number of individuals in HC, inactive, ANR and AR group were 18, 20, 20, and 28 for African Americans **(A)**, and 18, 19, 16, 20 for Caucasians **(B)**, respectively. Number of individuals in each group for Asians **(C)** was 20. In African-American **(A)**, Caucasian **(B)**, and Asian **(C)** individuals, urine sCD163 was significantly elevated in patients with active LN when compared with HC, or inactive SLE patients, or even ANR. In African-American patients, urine sCD163 also discriminated ANR or inactive from HC. ROC analysis showed urine sCD163 significantly discriminated active LN patients from HC, inactive SLE and ANR. Urine sCD163 was significantly correlated with SLEDAI and rSLEDAI, irrespective of ethnicities, and it strongly correlated with PGA in African Americans and Asians. R, Spearman’s correlation coefficient; HC, healthy control; ANR, active non-renal lupus patient; AR, active renal lupus; PGA, physician global assessment; ^∗^*P* < 0.05, ^∗∗^*P* < 0.01, ^∗∗∗^*P* < 0.001, ^*⁣*⁣**^P < 0.0001.

The primary cohort from JHU was comprised of 48 active LN, 36 ANR, 39 inactive SLE patients, and 36 healthy controls (Table 1). Analyses were done separately for African Americans and Caucasians. Results demonstrated that in both African Americans and Caucasians, Cr normalized urine sCD163 was significantly elevated in patients with active LN when compared with healthy controls, or inactive SLE, or ANR patients (all *P* < 0.001). In African American patients, urine sCD163 could further discriminate ANR or inactive patients from healthy controls (both *P* < 0.05). Importantly, urine sCD163 significantly correlated with SLEDAI and rSLEDAI in both the African American and Caucasian cohorts, and correlated strongly with PGA in African American subjects (Figures 1A,B).

**TABLE 1:** Characteristics of primary cohort with African American and Caucasian patients.

		**HC**	**Inactive SLE**	**Active non-renal**	**Active LN**
		***n* = 36**	***n* = 39**	***n* = 36**	***n* = 48**
**Ethnicity**					
	African American, *n* (%)	18 (50.00%)	20 (51.28%)	20 (55.56%)	28 (58.33%)
	Caucasian, *n* (%)	18 (50.00%)	19 (48.72%)	16 (44.44%)	20 (41.67%)
Female, *n* (%)		32 (88.89%)	35 (89.74%)	32 (88.89%)	44 (91.67%)
Age (years)		30.93 ± 5.34	38.96 ± 12.10	39.86 ± 11.88	34.08 ± 10.60
**Clinical assessment**					
	SLEDAI	N/A	0.64 ± 0.93	7.00 ± 2.16	8.02 ± 3.86
	rSLEDAI	N/A	0	0	5.42 ± 2.54
	PGA	N/A	0.70 ± 0.73	1.17 ± 0.68	1.79 ± 0.58
**System involvement**					
	Mucocutaneous, *n* (%)	N/A	6 (15.38%)	32 (88.89%)	12 (25.00%)
	Joints, *n* (%)	N/A	5 (12.82%)	11 (30.56%)	5 (10.42%)
	Neurological, *n* (%)	N/A	0 (0.00%)	2 (5.56%)	0 (0.00%)
	Hematological, *n* (%)	N/A	7 (17.95%)	5 (13.89%)	2 (4.17%)
**Laboratory measurement**					
	uPr/Cr (mg/mg)	N/A	0.17 ± 0.13	0.11 ± 0.09	2.01 ± 2.19
	ESR (mm/h)	N/A	28.60 ± 29.88	41.26 ± 24.09	39.50 ± 28.93
	SCr (mg/dl)	N/A	0.84 ± 0.30	0.82 ± 0.19	0.90 ± 0.30
	anti-dsDNA positivity, *n* (%)	N/A	4 (10.26%)	29 (80.56%)	25 (52.08%)
	anti-dsDNA titer (IU/ml)	N/A	12.84 ± 49.18	143.11 ± 209.05	95.44 ± 170.15
	C3 (mg/dl)	N/A	115.44 ± 26.51	80.72 ± 29.53	93.79 ± 31.62
	C4 (mg/dl)	N/A	20.88 ± 7.54	13.56 ± 7.74	18.77 ± 10.61
**Medications**					
	Prednisone, *n* (%)*	N/A	15 (38.46%)	19 (52.78%)	32 (66.67%)
	Hydroxychloroquine, *n* (%)	N/A	32 (82.05%)	31 (86.11%)	38 (79.17%)
	Mycophenolate mofetil, *n* (%)	N/A	17 (43.59%)	20 (55.56%)	29 (60.42%)
	Azathioprine, *n* (%)	N/A	3 (7.69%)	3 (8.33%)	6 (12.50%)
	Tacrolimus, *n* (%)	N/A	0 (0.00%)	2 (5.56%)	2 (4.17%)
	Cyclophosphamide, *n* (%)	N/A	0 (0.00%)	0 (0.00%)	1 (2.08%)
	Methotrexate, *n* (%)	N/A	1 (2.56%)	3 (8.33%)	1 (2.08%)
**Urine sCD163 (pg/ml)/(mg/dl)**		0 (0, 0)	0 (0, 0.88)	0 (0, 0.04)	5.56 (1.50, 13.41)
	African American	0 (0, 0)	0 (0, 1.28)	0 (0, 0.61)	9.23 (2.96, 26.22)
	Caucasian	0 (0, 0)	0 (0, 0)	0 (0, 0)	3.22 (0.55, 5.88)

Data were presented as numbers (percentage), average ± standard deviation, or median (Q1, Q3). *Percentage of patients taking prednisone was significantly different between inactive SLE and active LN. *P* < 0.05.

### Urine sCD163 Was Also Significantly Elevated in Active LN in Asian Patients

We further validated urine sCD163 in another cohort of patients, comprised of 20 active LN, 20 ANR, 20 inactive SLE, and 20 healthy controls, all of Asian origin (Table 2). In Asian patients, urine sCD163 was also significantly elevated in active LN compared with other SLE patients or healthy controls, and it was strongly correlated with SLEDAI, rSLEDAI, and PGA (Figure 1C). Notably, in patients with active LN, when three ethnic groups were compared, urine sCD163 was higher in African-American and Asian subjects than in Caucasian patients.

**TABLE 2:** Characteristics of secondary validation cohort with Asian patients.

		**HC**	**Inactive SLE**	**Active non-renal**	**Active LN**
		***n* = 20**	***n* = 20**	***n* = 20**	***n* = 20**
Female, *n* (%)		20 (100.00%)	19 (95.00%)	19 (95.00%)	19 (95.00%)
Age (years)		24.58 ± 3.78	45.10 ± 11.25	33.5 ± 14.28	38.30 ± 12.28
**Clinical assessment**					
	SLEDAI	N/A	2.45 ± 1.61	9.25 ± 6.63	15.05 ± 4.80
	rSLEDAI	N/A	0	0	8.20 ± 3.55
	PGA	N/A	0.17 ± 0.088	1.84 ± 0.32	2.14 ± 0.25
**System involvement**					
	Mucocutaneous, *n* (%)	N/A	0 (0.00%)	14 (70.00%)	8 (40.00%)
	Joints, *n* (%)	N/A	0 (0.00%)	7 (35.00%)	7 (35.00%)
	Neurological, *n* (%)	N/A	0 (0.00%)	1 (5.00%)	0 (0.00%)
	Hematological, *n* (%)	N/A	1 (5.00%)	10 (50.00%)	3 (15.00%)
**Laboratory measurement**					
	uPr/Cr (mg/mg)	N/A	N/A	N/A	2.73 ± 2.28
	anti-dsDNA positivity, *n* (%)	N/A	11 (55.00%)	14 (70.00%)	17 (85.00%)
	anti-dsDNA titer (IU/ml)	N/A	101.00 ± 82.98	159.05 ± 122.17	222.25 ± 99.06
	C3 (mg/dl)	N/A	89.45 ± 28.03	75.90 ± 27.00	47.75 ± 23.14
	C4 (mg/dl)	N/A	16.95 ± 8.16	12.35 ± 5.73	8.90 ± 8.39
**Medications**					
	Prednisone, *n* (%)	N/A	18 (90.00%)	20 (100.00%)	20 (100.00%)
	Hydroxychloroquine, *n* (%)*	N/A	12 (60.00%)	19 (95.00%)	14 (70.00%)
	Mycophenolate mofetil, *n* (%)*	N/A	4 (20.00%)	4 (20.00%)	15 (75.00%)
	Azathioprine, *n* (%)	N/A	17 (85.00%)	12 (60.00%)	17 (85.00%)
	Tacrolimus, *n* (%)	N/A	2 (10.00%)	3 (15.00%)	6 (30.00%)
	Cyclophosphamide, *n* (%)	N/A	6 (30.00%)	4 (20.00%)	7 (35.00%)
	Cyclosporin, *n* (%)	N/A	3 (15.00%)	2 (10.00%)	2 (10.00%)
**Urine sCD163 (pg/ml)/(mg/dl)**		0 (0, 0)	0 (0, 0.76)	0 (0, 1.41)	22.02 (7.97, 32.78)

Data were presented as numbers (percentage), average ± standard deviation, or median (Q1, Q3). *Percentage of patients taking hydroxychloroquine was significantly higher in active non-renal group; percentage of patients taking mycophenolate mofetil was significantly higher in active LN group. *P* < 0.05.

### Urine sCD163 Was Significantly Elevated in the Presence of Concurrent Proliferative LN

**FIGURE 2:**
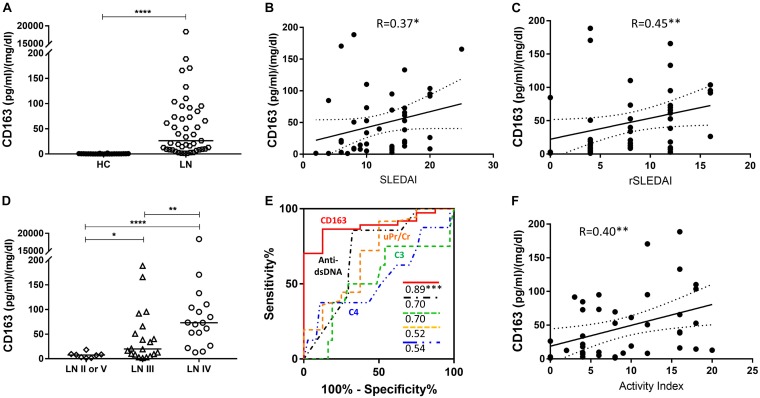
Urine sCD163 in a LN cohort with concurrent renal biopsies. **(A)**, Urine sCD163 was significantly elevated in patients with LN (*n* = 45) when compared with HC (*n* = 24). The horizontal bar represents the median. **(B,C)** Urine sCD163 was significantly correlated with SLEDAI and rSLEDAI. One outliner is not displayed in the correlation plots. **(D)** Urine sCD163 was significantly elevated in patients with LN IV (*n* = 17) when compared with those with LN III (*n* = 20) or LN II or LN V (*n* = 8). For this analysis, patients with Class III + V and IV + V LN were categorized as Class III or IV, respectively. **(E)** Urine sCD163 outperformed C3, C4, uPr/Cr, or anti-dsDNA antibody in discriminating LN II or V from LN III or IV. Values in the plot indicate areas under curve. **(F)** Urine sCD163 was significantly correlated with activity index (AI) of renal pathology. R, Spearman’s correlation coefficient; HC, healthy control; LN, lupus nephritis; uPr/Cr, urine protein to creatinine ratio; ^∗^*P* < 0.05, ^∗∗^*P* < 0.01, ^∗∗∗^*P* < 0.001, ^*⁣*⁣**^P < 0.0001.

Given that urine sCD163 was elevated in active LN patients, we further explored whether the elevation of urine sCD163 differed between LN classes. Urine sCD163 was assayed in 45 LN patients with concurrent renal biopsies (Table 3). This cohort yielded matched urine/renal-tissue specimens from the same patients, allowing one to examine if urine proteins could predict concurrent renal pathology changes. Patients were dichotomized as proliferative LN (class III or IV, *n* = 37) or non-proliferative LN (class II or V, *n* = 8), based on pathology analysis of the biopsy. Among the renal biopsies from patients with Class III or IV LN, the frequencies of AI-related pathological changes were as follows: endocapillary hypercellularity, 77.14%; glomerular leukocyte infiltration, 57.14%; wire loop deposits, 42.86%; fibrinois necrosis, 48.57%; cellular crescents, 62.86%; interstitial inflammation, 62.86%. Likewise, in these biopsies, the frequencies of CI-related pathological attributes were as follows: glomerulosclerosis, 62.86%; fibrous crescents, 11.43%; tubular atrophy and interstitial fibrosis, 62.86%. Urine sCD163 was significantly elevated in patients with proliferative LN, especially in LN IV, and it outperformed conventional parameters including C3, C4, and anti-dsDNA antibody in differentiating proliferative LN from non-proliferative diseases (Figures 2A–E).

**TABLE 3:** Characteristics of LN patients with concurrent renal biopsies.

		**All LN**	**LN II or V**	**LN III or IV**	***P* value^‡^**
		***n* = 45**	***n* = 8**	***n* = 37**	
**Race**					
	African American, *n* (%)	17 (37.78%)	3 (37.50%)	14 (37.84%)	>0.9999
	Caucasian, *n* (%)	24 (53.33%)	5 (62.50%)	19 (51.35%)	0.705
	Asian, *n* (%)	4 (8.89%)	0 (0.00%)	4 (10.81%)	>0.9999
**Female, *n* (%)**		41 (91.11%)	6 (75.00%)	35 (94.59%)	0.1395
**Age (years)**		31.58 ± 8.82	30.63 ± 6.37	31.78 ± 9.32	0.8787
**Clinical assessment**					
	SLEDAI	12.71 ± 4.85	10.25 ± 5.92	13.24 ± 4.50	0.1299
	rSLEDAI	8.53 ± 4.14	6.00 ± 4.28	9.08 ± 3.96	0.0728
**Laboratory measurement**					
	uPr/Cr (mg/mg)	3.27 ± 2.65	2.05 ± 2.18	3.54 ± 2.69	0.0842
	Scr (mg/dl)	1.68 ± 1.61	1.78 ± 1.76	1.66 ± 1.60	0.3971
	anti-dsDNA positivity, *n* (%)	30 (66.67%)	4 (50.00%)	26 (70.27%)	0.2019
	anti-dsDNA titer (IU/ml)	853.00 ± 1013.62	172.50 ± 311.70	957.69 ± 1046.26	0.1638
	C3 (mg/dl)	76.24 ± 35.74	77.63 ± 38.40	75.95 ± 35.69	0.8884
	C4 (mg/dl)	11.60 ± 10.79	10.13 ± 7.04	11.92 ± 11.49	0.9666
**Renal pathology^§^**					
	Activity index	8.90 ± 5.83	3.57 ± 3.05	9.97 ± 5.69	0.0048**
	Endocapillary hypercellularity, *n* (%)	28 (66.67%)	1 (14.29%)	27 (77.14%)	0.0032**
	Glomerular leukocyte infiltration, *n* (%)	21 (50.00%)	1 (14.29%)	20 (57.14%)	0.0931
	Wire loop deposits, *n* (%)	20 (47.62%)	5 (71.43%)	15 (42.86%)	0.2289
	Fibrinoid necrosis, *n* (%)	17 (40.48%)	0 (0.00%)	17 (48.57%)	0.03*
	Cellular crescents, *n* (%)	22 (52.38%)	0 (0.00%)	22 (62.86%)	0.0029**
	Interstitial inflammation, *n* (%)	23 (54.76%)	1 (14.29%)	22 (62.86%)	0.0341*
	Chronicity index	3.93 ± 3.01	4.57 ± 3.21	3.80 ± 3.00	0.5975
	Glomerulosclerosis, *n* (%)	27 (64.29%)	5 (71.43%)	22 (62.86%)	>0.9999
	Fibrous crescents, *n* (%)	5 (11.90%)	1 (14.29%)	4 (11.43%)	>0.9999
	Tubular atrophy and interstitial fibrosis, *n* (%)	26 (61.90%)	4 (57.14%)	22 (62.86%)	>0.9999
**Medications**					
	Prednisone, *n* (%)	45 (100.00%)	8 (100.00%)	37 (100.00%)	>0.9999
	Hydroxychloroquine, *n* (%)	40 (88.89%)	7 (87.50%)	33 (89.19%)	>0.9999
	Mycophenolate mofetil, *n* (%)	35 (77.78%)	4 (50.00%)	31 (83.78%)	0.1383
	Azathioprine, *n* (%)	4 (8.89%)	0 (0.00%)	4 (10.81%)	>0.9999
	Cyclosporin, *n* (%)	1 (2.22%)	0 (0.00%)	1 (2.70%)	>0.9999
	Cyclophosphamide, *n* (%)	1 (2.22%)	0 (0.00%)	1 (2.70%)	>0.9999
**Urine sCD163 (pg/ml)/(mg/dl)**		26.34 (9.42, 73.42)	7.43 (2.70, 8.85)	50.77 (14.54, 91.71)	0.0002

*Data were presented as numbers (percentage), average ± standard deviation, or median (Q1, Q3). ‡*P* values were for comparisons between LN II or V and LN III or IV. §In the renal pathology section, the percentages were calculated based on the number of patients who had complete information for AI and CI (all LN, *n* = 42; LN II or V, *n* = 7; LN III or IV, *n* = 35).*

### Urine sCD163 Significantly Correlated With Renal Pathological Activity Indices

Given that urine sCD163 was significantly elevated in patients with concurrent proliferative LN when compared with non-proliferative LN, we then investigated whether it correlated with particular pathological attributes. Importantly, urine sCD163 significantly correlated with concurrent AI of renal pathology (Figure 2F), particularly with fibrinoid necrosis, cellular crescents, and interstitial inflammation. In contrast, conventional parameters including C3, C4, and anti-dsDNA antibody did not correlate with AI or its component attributes. However, urine sCD163 did not correlate with renal pathology chronicity index (CI) or its component histological attributes (Table 4).

**TABLE 4:** Correlation of urine sCD163 and conventional metrics with renal pathology activity and chronicity indices in biopsy-concurrent LN patients.

	**AI**	**Endocapillary hypercellularity**	**Glomerular leukocyte infiltration**	**Wire loop deposits**	**Fibrinoid necrosis**	**Cellular crescents**	**Interstitial inflammation**	**CI**	**Glomerulo-sclerosis**	**Fibrous crescents**	**Tubular atrophy and interstitial fibrosis**
CD163	0.40**	0.18	0.20	−0.22	0.45**	0.48**	0.39*	−0.14	−0.08	−0.02	0.05
C3	−0.19	−0.22	−0.10	−0.06	−0.13	−0.05	−0.21	0.28	0.46**	0.32*	−0.07
C4	−0.08	−0.11	−0.08	−0.02	0.004	−0.004	−0.25	0.28	0.41**	0.13	−0.01
anti-dsDNA	0.094	0.19	−0.078	−0.11	0.12	0.060	−0.071	−0.29	−0.41**	−0.26	−0.12

Values in the table indicate *R* values by Spearman correlation analysis. AI, activity index; CI, chronicity index; *P < 0.05; **P < 0.01.

**FIGURE 3:**
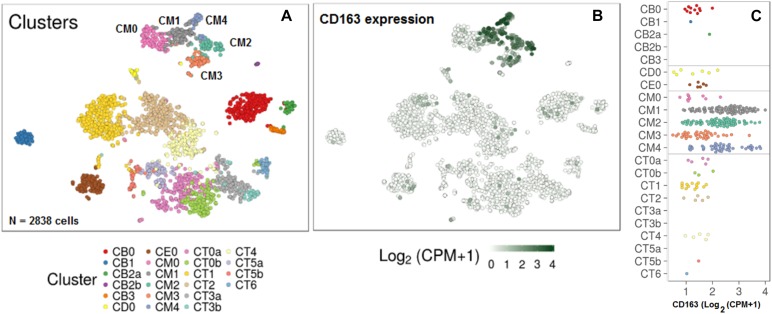
Maximal expression of CD163 within infiltrating M2 macrophages in kidneys of 24 patients with lupus nephritis. **(A)** Clusters of kidney cells identified with public scRNA-seq data. Cluster annotations: CM0, CD16 + macrophages, inflammatory; CM1, CD16 + macrophages, phagocytic; CM2, tissue-resident macrophages; CM3, cDCS; CM4, CD16 + macrophages, M2-like; CT0a, effector memory CD4 + T cells; CT0b, central memory CD4 + T cells; CT1, CD56^dim^ CD16 + NK cells; CT2, CTLs; CT3a, Tregs; CT3b, TFH-like cells; CT4, GZMK + CD8 + T cells; CT5a, resident memory CD8 + T cells; CT5b, CD56^bright^ CD16- NK cells; CT6, ISG^high^ CD4 + T cells; CB0, activated B cells; CB1, plasma cells/plasmablasts; CB2a, naïve B cells; CB2b, pDCs; CB3, ISG^high^ B cells; CD0, dividing cells; CE0, epithelial cells. **(B,C)** CD163 expression on kidney cells, predominantly on macrophages.

Urine sCD163 could potentially be derived from intra-renal infiltrating immune cells, given that myeloid cells are known to express this surface molecule. Single cell RNA-sequencing data (scRNA-seq) of renal infiltrating immune cells have recently been reported (14). Interrogation of this publicly deposited database indicated that macrophages were the predominant source of surface CD163 within LN kidneys, particularly M2 macrophages (Figures 3A–C), although CD163 was also expressed on other intra-renal macrophage populations including phagocytic CD16^+^ macrophages and tissue-resident macrophages. These findings suggest that intra-renal macrophages, particularly the M2 macrophages, may be the dominant source of sCD163 in the urine of LN patients.

## Discussion

Conventional biomarkers, including C3, C4, and anti-dsDNA antibody, have been classicly used to evaluate general disease activity in SLE. However, they do not predict or correlate well with LN or disease flares (8, 15). Anti-C1q is associated with renal involvement in SLE patients, particularly proliferative glomerulonephritis (16). Urinary biomarkers have now emerged as a potential tool for evaluating LN and potential treatment targets, as these proteins may arise directly from the inflamed kidneys (8, 15). Several newly identified urine biomarkers including monocyte chemoattractant protein-1 (MCP-1), neutrophil gelatinase -B associated lipocalin (NGAL), TNF-like WEAK inducer of apoptosis (TWEAK), and vascular cell adhesion molecule-1 (VCAM), have been promising in monitoring disease status (8). The identification of better biomarkers which significantly discriminate active LN and strongly correlate with renal biopsy or underlying mechanisms has been a priority.

We found that urine sCD163 significantly correlated with conventional parameters including urine protein to creatinine ratio, anti-dsDNA and serum C3 (all *P* < 0.05, Supplementary Table 1), and outperformed C3, C4, urine protein to creatinine ratio, and anti-dsDNA antibody in discriminating proliferative LN from non-proliferative LN (Figure 2E). Its strong correlation with concurrent renal pathology activity further supports the further study of urine sCD163 as a promising biomarker of LN.

This predictive potential of sCD163 may be attributed to the close connection of urine sCD163 with disease pathogenesis. CD163, a transmembrane scavenger receptor, is exclusively expressed on macrophages and monocytes (17). Macrophages have been implicated in pathogenesis of SLE in many studies, with the spectrum of activation phenotypes ranging from classically activated inflammatory M1 to alternatively activated M2 macrophages (18–20). M2 macrophages can be further classified as M2a, M2b and M2c, which display distinct functions of pro-fibrosis, immunity-regulation, and remodeling or anti-inflammation, respectively (19). CD163 has been recognized as a marker of M2 macrophages, especially M2c (21). These findings are clearly consistent with recent scRNA-seq analysis, revealing a predominant CD163^high^ M2 macrophages in LN kidneys (Figures 3A–C) (14).

It has been reported that M1 macrophages are dominant in SLE, as suggested by elevated markers of M1 macrophages, the cytokine milieu favoring M1 macrophages, and predisposing genetic factors, while M2a and M2c subpopulations were reduced in SLE, potentially contributing to defective anti-inflammatory balance (18–20). However, the predominant intra-renal subpopulations of macrophages in SLE have been controversial and vary across studies, partly due to the markers used for identification of subpopulations. In one study, immunohistochemistry (IHC) demonstrated that infiltration of macrophages was dominated by CD163^+^ M2c-like macrophages in both glomerular and tubulointerstitial compartments (22). CD163^+^ M2c-like macrophages were significantly elevated in LN III and LN IV when compared with LN V, and correlated with AI of renal pathology (22). While tubulointerstitial CD163^+^ macrophages significantly correlated with serum creatinine, serum urea and creatinine clearance, glomerular CD163^+^ macrophages negatively correlated with plasma C3 and C4 (22, 23). Additionally, CD163 was found mainly expressed in active crescentic glomerulonephritis, proliferative glomerular lesions and areas of tubulointerstitial injury (21–24). In another study including patients with pauci-immune necrotizing glomerulitis, CD68^+^ and CD163^+^ macrophages predominated at sites of fibrinoid necrosis (25). These literature reports, taken together with our findings in concurrent renal biopsies, suggest that M2 macrophages may play an important role in driving or modulating interstitial inflammation, cellular crescent formation, and fibrinoid necrosis. Furthermore, the predominant expression of CD163 by M2 macrophages rather than other renal cells supports the use of urine sCD163 as an easily measurable yardstick of renal macrophage infiltration.

CD163 expression on macrophages has been reported to be influenced by several medications including glucocorticoids, mycophenolate mofetil (MMF), tacrolimus, rituximab, and cyclophosphamide (22, 26–29). In the primary cohort with African American and Caucasian patients, the percentage of patients taking prednisone was significantly higher in active LN compared to the inactive group (Table 1). In the validation cohort with Asian patients, the percentage of patients taking MMF was significantly higher in active LN group compared to inactive or ANR group (Table 2). However, in each ethnic group, when patients who took a certain medication were compared with patients who did not take the same medication, no significant difference in urine sCD163 was noted (Supplementary Table 2). Thus, the observed elevation in urine sCD163 in active LN and proliferative LN could not be attributed to medications. Indeed, it has been reported that urine sCD163 levels were comparable between glucocorticoid treated and untreated patients with LN IV, and was not related to the dosage of glucocorticoids (23).

Whether other organ involvement in SLE could influence the level of urine sCD163 has been questioned. Of relevance, serum/plasma level of sCD163 was significantly higher in SLE compared to healthy controls, and it correlated with anti-dsDNA antibodies, anti-chromatin antibodies, leukopenia, and SLEDAI (9, 30, 31). However, elevated plasma sCD163 levels in LN were not associated with ISN/RPS class (23), suggesting that increased urine sCD163 is unlikely to be the consequence of enhanced leakage of circulating sCD163 or systemic activation of macrophages, although this needs to be examined more systematically.

Given that urine sCD163 is predictive of concurrent proliferative LN in patients with clinically active renal disease, and given its association with active crescent formation and interstitial inflammation, monitoring urine sCD163 might represent a convenient, non-invasive method to track underlying renal disease in patients with LN. Hence, urine sCD163 may potentially be useful to guide management of LN. Since proliferative LN and elevated renal pathology AI are associated with worse patient and renal outcome, aggressive management is warranted in these patients (32, 33). Further studies are clearly imperative to ascertain how urine sCD163 varies over time in the same patients, when serially monitored. Longitudinal studies are needed to assess if urine sCD163 can be used to predict renal flares or track response to treatment.

Elevated interleukin IL-6, IL-10, and macrophage colony-stimulating factor (M-CSF) in the glomerular microenvironment of LN have been reported to promote macrophage differentiation into CD163^+^ cells (23, 34). Nevertheless, the origin of increased macrophages in LN is not clear. One study suggested that tissue-resident macrophages and circulating monocytes were independently maintained, and monocytes did not contribute significantly to tissue macrophages in the steady state (35). However, others have proposed that resident macrophages are dominant in kidneys and are distinct from infiltrating macrophages that originate and renew from bone marrow, and that resident and infiltrating inflammatory macrophages both contribute to ongoing renal damage (36, 37). Moreover, the role of macrophages as well as CD163^+^ cells in LN has not been fully elucidated. In several mouse LN models, systemic depletion of macrophages or inhibition of macrophage recruitment ameliorated nephritis (20), while in others, depletion of macrophages slowed resolution and promoted fibrosis (37, 38). M2c macrophages are considered to have remodeling or anti-inflammatory roles (19, 22). Polarization of macrophages to a M2c-like phenotype is essential for efficient clearance of apoptotic cells, which when defective contribute to initiation and perpetuation of SLE, making induction of M2 macrophages an attractive therapy for SLE (39, 40). However, given the increased infiltration of CD163^+^ macrophages in active crescents, proliferative LN and acute tubulointerstitial lesions, it has also been suggested that CD163^+^ macrophages are involved in disease progression in kidney injury (24). Targeting macrophages has been suggested as a promising SLE therapeutic strategy (20), but whether CD163 would serve as a more precise target in modulating macrophages needs to be investigated.

To conclude, urine sCD163 was significantly elevated in active LN. It accurately discriminated proliferative LN from non-proliferative LN and strongly correlated with concurrent renal pathological AI as well as fibrinoid necrosis, cellular crescents, and interstitial inflammation. These features support the use of urine sCD163 as a measurable biomarker for evaluating renal disease progression and for guiding management of LN. Prospective studies in larger cohorts to evaluate the performance of urine sCD163 as well as in-depth mechanistic studies unraveling the role of CD163 in LN pathogenesis are warranted.

## Data Availability Statement

The datasets generated for this study are available on request to the corresponding author.

## Ethics Statement

This study was approved by the Institutional Review Board of the University of Houston, JHU School of Medicine, Tuen Mun Hospital, and UTSW.

## Author Contributions

CM designed the study. CCM, MP, and RS collected the samples used in this study. TZ, HL, and KV performed the experiment. TZ, GG, and CM analyzed the data. TZ, CCM, MP, RS, and CM drafted and revised the manuscript. All authors approved the final version of the manuscript.

## Conflict of Interest

The authors declare that the research was conducted in the absence of any commercial or financial relationships that could be construed as a potential conflict of interest.
