# Supplementary material for: Association of Urine sCD163 With Proliferative Lupus Nephritis, Fibrinoid Necrosis, Cellular Crescents and Intrarenal M2 Macrophages
Source: Front Immunol. 2020 Apr 15;11:671. doi: 10.3389/fimmu.2020.00671 (PMC7174755; doi:10.3389/fimmu.2020.00671)
Supplement: Supplementary file 1 [file Image_1.PDF]

Supplementary Figure

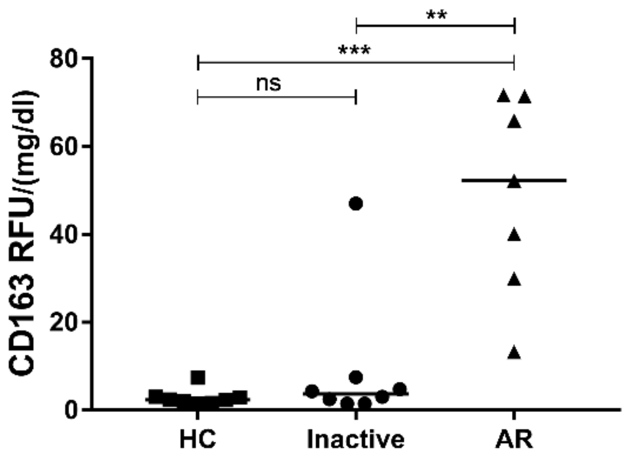

Supplementary Figure 1. Urine sCD163 levels in preliminary screening. Urine sCD163 was significantly elevated in patients with active renal lupus when compared with healthy controls, or inactive SLE patients. Number of individuals in each group was eight. HC, healthy controls; AR, active renal lupus; *, *P* <0.05; **, *P* <0.01; ***, *P* <0.001; ****, *P* <0.0001.
