# Supplementary material for: Association of Urine sCD163 With Proliferative Lupus Nephritis, Fibrinoid Necrosis, Cellular Crescents and Intrarenal M2 Macrophages
Source: Front Immunol. 2020 Apr 15;11:671. doi: 10.3389/fimmu.2020.00671 (PMC7174755; doi:10.3389/fimmu.2020.00671)
Supplement: Supplementary file 2 [file Table_1.DOCX]

**Supplementary Tables**

| **Supplementary Table 1.** Correlation of urine sCD163 and conventional metrics in biopsy-concurrent LN patients | | | | | | | | | |
| --- | --- | --- | --- | --- | --- | --- | --- | --- | --- |
|  | SLICC | rSLICC | uPr/Cr | Serum Cr | C3 | C4 | CRP | anti-dsDNA | ANA titer |
| CD163 | -0.068 | -0.039 | 0.47** | -0.018 | -0.40** | -0.24 | 0.19 | 0.34* | -0.13 |
| R values from Spearman correlation analysis were demonstrated. SLICC, Systemic Lupus International Collaborating Clinics; uPr/Cr, urine protein to creatinine ratio; Cr, creatinine; CRP, C-reactive protein; *, *P* <0.05; **, *P* <0.01. | | | | | | | | | |

| **Supplementary Table 2.** Comparison of urine sCD163 in SLE patients with or without a medication | | | |
| --- | --- | --- | --- |
|  | African-American | Caucasian | Asian |
| Prednisone | 0.1306 | 0.2963 | N/A |
| Hydroxychloroquine | 0.7928 | 0.4786 | 0.6015 |
| Mycophenolate mophetil | 0.3531 | 0.5002 | 0.3486 |
| Azathioprine | 0.358 | 0.6159 | 0.6895 |
| Tacrolimus | N/A | 0.8535 | 0.968 |
| Cyclophosphamide | 1 | N/A | 0.06749 |
| Methotrexate | 0.4576 | N/A | N/A |
| Cyclosporin | N/A | N/A | 0.5895 |
| Values demonstrated in the table are *P* values calculated with Mann-Whitney test. | | | |
